# Monocyte-to-lymphocyte ratio and further inflammatory parameters as potential biomarkers of suicide risk in bipolar affective disorder

**DOI:** 10.1192/j.eurpsy.2025.724

**Published:** 2025-08-26

**Authors:** B. Pethő, T. Tényi, R. Herold, D. Simon, T. Tóth, C. Molnár, M. Á. Kovács

**Affiliations:** 1 Department of Psychiatry and Psychotherapy; 2 Department of Immunology and Biotechnology; 3 1st Department of Internal Medicine, University of Pécs, Clinical Centre, Pécs, Hungary

## Abstract

**Introduction:**

Suicide is an unresolved issue in psychiatry to this day. Suicide risk (SR) is highest for psychiatric patients with bipolar affective disorder (BD). Recent studies suggest an immunological dysregulation in the background of suicidality, and in accord with former results, our research group has previously found monocyte-to-lymphocyte ratio (MLR) and further inflammatory parameters to be reliable indicators of SR in patients with major depressive disorder.

**Objectives:**

Determining SR remains a challenge for clinicians. Alterations in the number and ratio of inflammatory cells have been proposed as potential biomarkers of SR, therefore our aim was to investigate changes of these parameters in relation to acute and long-term SR in BD patients.

**Methods:**

In our restrospective study, we investigated laboratory parameters of psychiatric inpatients diagnosed with BD between January 2020 and June 2024. Data was collected regarding the following parameters: white blood cell, neutrophil, lymphocyte, monocyte and platelet count, MLR, neutrophil-to-lymphocyte (NLR) and platelet-to-lymphocyte ratio (PLR), C-reactive protein (CRP), erythrocyte sedimentation rate (ESR), red blood cell distribution width (RDW) and mean platelet volume (MPV). Individuals with recent (≤ 48 hours prior) suicide attempt (SA) (*n* = 21) and with past (> 48 hours prior) SA (*n* = 16) represented the high SR group (*n* = 37). BD patients with no history of SA (*n* = 79) composed the intermediate SR group. Statistical analyses were carried out using the GraphPad Prism 10.3.1 programme.

**Results:**

We found a significant increase in MLR (*p* = 0.0021, Fig. 1), monocyte count (*p* = 0.0045), CRP (*p* = 0.0036), ESR (*p* ≤ 0.0001) and MPV (*p* ≤ 0.0001) in patients with recent SA compared to those with no history of SA. Comparing high and intermediate risk patients, MLR (*p* = 0.012, Fig. 2), monocyte count (*p* = 0.0293), CRP (*p* = 0.0499, Fig. 3), ESR (*p* = 0.0009) and MPV (*p* = 0.008) remained elevated in the former group. We found no significant differences regarding the rest of the parameters. According to ROC analysis, the probability of the significant results was outstanding in case of ESR and acceptable for the rest of the parameters.

**Image 1:**

**Figure:**
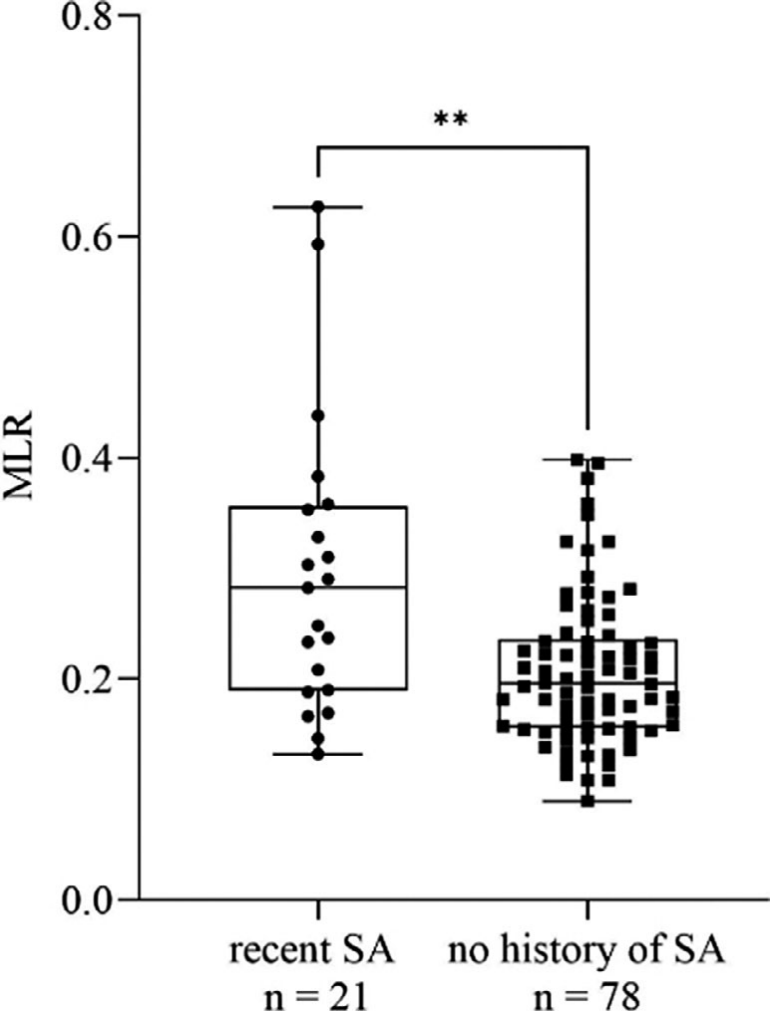

**Image 2:**

**Figure:**
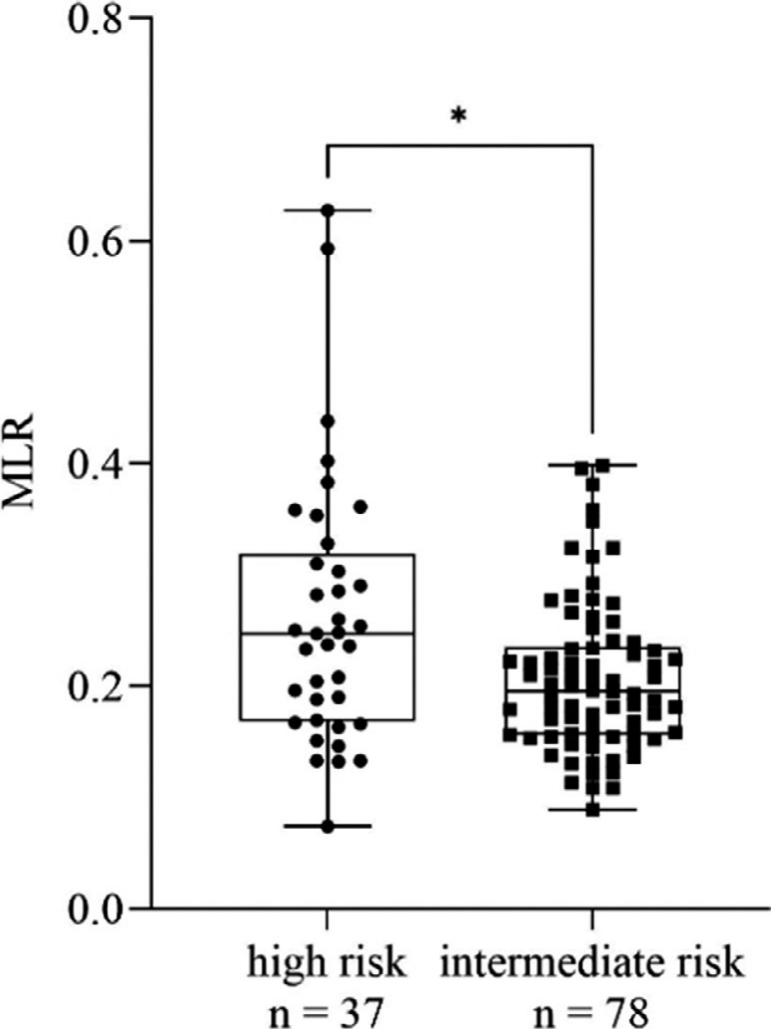

**Image 3:**

**Figure:**
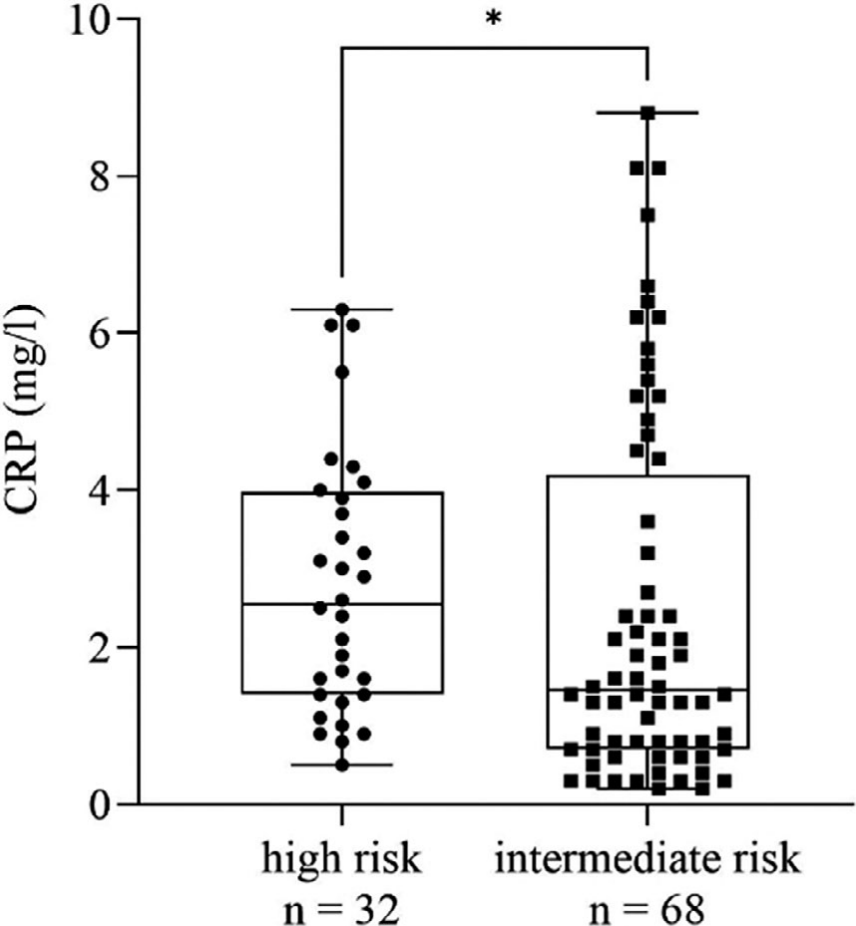

**Conclusions:**

As implied by previous research, immunological mechanisms may contribute to the emergence of suicidality. Investigating BD patients as the subgroup most at risk, we found MLR, monocyte count, CRP, ESR and MPV to show significant alterations in recent attempters, signalling acute SR. Furthermore, all of these markers were significantly elevated in high risk patients, therefore they may be indicative of long-term SR. Changes in these inflammatory markers further strengthen the assumption of immunological processes in the background of suicidality, and these parameters may serve as potential future biomarkers of SR.

The study was supported by the EKÖP-24-3-II-PTE-168 project.

**Disclosure of Interest:**

None Declared

